# High-throughput, low-cost FLASH: irradiation of *Drosophila melanogaster* with low-energy X-rays using time structures spanning conventional and ultrahigh dose rates

**DOI:** 10.1093/jrr/rrae079

**Published:** 2024-10-18

**Authors:** Alexander Hart, Jan P Dudzic, Jameson W Clarke, Jonathan Eby, Steve J Perlman, Magdalena Bazalova-Carter

**Affiliations:** Department of Physics and Astronomy, University of Victoria, 3800 Finnerty Road, Victoria, BC V8P 5C2, Canada; Department of Biology, University of Victoria, 3800 Finnerty Road, Victoria, BC V8P 5C2, Canada; Department of Biology, University of Victoria, 3800 Finnerty Road, Victoria, BC V8P 5C2, Canada; Institute of Biomedical Engineering, University of Toronto, 164 College St. Toronto, Ontario M5S 3E2, Canada; Department of Biology, University of Victoria, 3800 Finnerty Road, Victoria, BC V8P 5C2, Canada; Department of Physics and Astronomy, University of Victoria, 3800 Finnerty Road, Victoria, BC V8P 5C2, Canada

**Keywords:** ultrahigh dose-rate radiotherapy, UHDR, FLASH, *Drosophila*, low-energy X-rays, radiobiology

## Abstract

FLASH radiotherapy is an emerging technique in radiation oncology that may improve clinical outcomes by reducing normal tissue toxicities. The physical radiation characteristics needed to induce the radiobiological benefits of FLASH are still an active area of investigation. To determine the dose rate, range of doses and delivery time structure necessary to trigger the FLASH effect, *Drosophila melanogaster* were exposed to ultrahigh dose rate (UHDR) or conventional radiotherapy dose rate (CONV) 120-kVp X-rays. A conventional X-ray tube outfitted with a shutter system was used to deliver 17- to 44-Gy doses to third-instar *D. melanogaster* larvae at both UHDR (210 Gy/s) and CONV (0.2–0.4 Gy/s) dose rates. The larvae were then tracked through development to adulthood and scored for eclosion and lifespan. Larvae exposed to UHDR eclosed at higher rates and had longer median survival as adults compared to those treated with CONV at the same doses. Eclosion rates at 24 Gy were 68% higher for the UHDR group (*P* < 0.05). Median survival from 22 Gy was *>*22 days for UHDR and 17 days for CONV (*P* < 0.01). Two normal tissue-sparing effects were observed for *D. melanogaster* irradiated with UHDR 120-kVp X-rays. The effects appeared only at intermediate doses and may be useful in establishing the dose range over which the benefits of FLASH can be obtained. This work also demonstrates the usefulness of a high-throughput fruit fly model and a low-cost X-ray tube system for radiobiological FLASH research.

## INTRODUCTION

The goal of modern radiation oncology is two fold: to deliver a sufficient dose of ionizing radiation to a tumor in order to stop the proliferation of cancer cells and also to minimize harm to normal tissue. Failure to achieve the latter can result in serious side effects and potentially cause additional cancers in the future. The conventional solution to this problem is to increase the conformality of radiotherapy treatments. Through techniques including intensity-modulated radiotherapy [1], hadron therapies [2], and advances in the repeatability of patient positioning and image guidance [3], radiation dose distributions can be carefully sculpted to minimize radiation to healthy tissues. Physicists and radiation oncologists have focused on effectively reducing the physical margins of radiotherapy, but recently there has been renewed interest in the radiobiological differences between cancerous and healthy tissue [4]. Radiobiologists have turned their attention to dose rate as a potential tool to find differential biological responses. Many studies have suggested that delivering doses at so-called ultrahigh dose rates (UHDR, ≥40 Gy/s) may cause less damage to normal tissue than conventional dose rates (CONV, ∼0.1 Gy/s) while maintaining cancer cell kill efficacy [5, 6]. This has been termed the FLASH effect.

The biological mechanisms behind UHDR radiotherapy and the FLASH effect are still under active investigation, with hypotheses focusing on transient oxygen depletion [7, 8], sparing of circulating immune cells [9, 10], and differential DNA damage between tumor and normal tissues [11, 12]. Before treating human patients with UHDR radiation sources in hopes of benefiting from the FLASH effect, it is imperative to develop a robust radiobiological understanding of UHDR radiotherapy through animal studies. *Drosophila melanogaster* have been central to the study of radiobiology since before the development of the linear accelerators commonly used for radiotherapy. Herman Joseph Muller’s fruit fly experiments in 1926–27 led to his receiving the Nobel prize for the discovery of X-ray mutagenesis [13]. Research conducted by Muller’s students also contributed directly to the development of the linear-no-threshold model for radiation protection, which posits that there is no `safe' dose of ionizing radiation [14, 15]. This work has faced scrutiny [16] and some have even suggested that there may be benefits to low doses of radiation [17]. Dose-rate radiobiology using *D. melanogaster* models has recently gained attention, but the field has been limited to very low dose rates (on the order of CONV dose rates or lower). Low-dose-rate priming doses (0.2 Gy) have been shown to reduce DNA damage and impact gene expression [18]. Other groups have studied the effects of low-dose-rate background irradiation from cosmic rays by shielding *D. melanogaster* deep underground. These works have demonstrated a link between dose rate and genetic and phenotypic responses [19, 20].

Beyond their historical relevance to radiobiology, there is a compelling case to be made for radiobiological studies of *D. melanogaster* as an excellent model for human cancers. To date, the most influential works on the FLASH effect have included studies of mice, mini pigs, cats [5, 21] and the early clinical trials with human patients [22]. It remains a serious issue that these studies involve low numbers of subjects with high heterogeneity of results. Development of new high-throughput models, such as *Caenorhabditis elegans*, would be helpful to increase the statistical power of radiobiological studies [23]. *Drosophila melanogaster* is another such model that could provide greater statistics while also offering a genetic model with direct analogs to humans [24, 25]. Models for a number of human cancers have been developed for both larvae and adult *D. melanogaster*, which can be used to develop new therapies [26]. Additionally, there are significant logistical advantages to working with fruit flies: no research ethics board approval is required, and results with large numbers of flies at various life stages can be procured on short notice. The life cycle of *D. melanogaster* is ~10 days from egg to emerged adult fly.

The irradiations described in this work were performed using a low-cost system based around a conventional X-ray tube [27]. FLASH research is typically an expensive undertaking, with most studies utilizing proton cyclotrons [28, 29] and experimental electron linear accelerators [30, 31]. The work described here seeks to demonstrate the viability and utility of a low-cost irradiation platform coupled with a high-throughput whole organism. By irradiating late-third-instar *D. melanogaster* larvae with 120-kVp X-rays at UHDR and conventional dose rates, we can gain insights about the range of doses and time structure of UHDR beams that are necessary to elicit the FLASH effect.

## MATERIALS AND METHODS

### Flies

Wild-type *D. melanogaster* (Oregon-R) were used in this study. Post irradiation, larvae were placed in a fresh vial with an antifungal grape juice medium (25 ml grape juice, 75 ml water, 2.25 g agar, 0.15 g nipogin) and kept at 24°C. After emergence, adult flies were placed in Carolina Instant *D. melanogaster* Medium and flipped twice weekly. The incubator was set to a 12-h light and dark cycle.

### Irradiation conditions

**Fig. 1:**
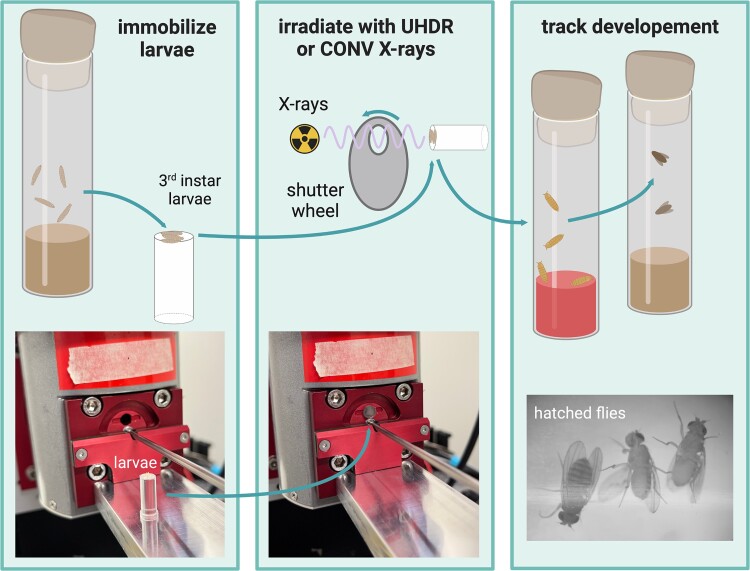
Procedure for immobilizing and irradiating third-instar larvae *D. melanogaster* within the X-ray tube shutter system. Post irradiation, larvae were placed in an antifungal medium while developing to pupae and adult flies.

Late-third-instar larvae were immobilized with double-sided tape on acrylic cylinders for the irradiations. Control groups were given a mock irradiation (immobilized but not exposed to X-rays). Three larvae were immobilized and irradiated simultaneously as shown in Fig. 1. For each experiment, at least 20 larvae were irradiated. Each experiment was repeated in triplicate on different days, for a total of 60 larvae irradiated per each dose and dose-rate group.

### Survival analysis

Flies were scored for eclosion (complete emergence of adults from pupae) starting at 4 days post irradiation. Fractional eclosion for each dose group (eclosed adults/irradiated larvae) was normalized to the percent eclosion from the control group treated on the same day such that:


$$ \%\mathrm{eclosion}=\frac{\mathrm{irradiated}\ \mathrm{eclosion}\ \mathrm{rate}}{\mathrm{control}\ \mathrm{eclosion}\ \mathrm{rate}}\times 100\% $$

Adult flies were also tracked for survival up to 22 days post irradiation. Deaths were scored once daily and flies lost or damaged during transfer were right-censored. After flipping adults into new vials, the previous vials were kept in order to check for offspring production.

### X-Ray system and dosimetry

**Fig. 2:**
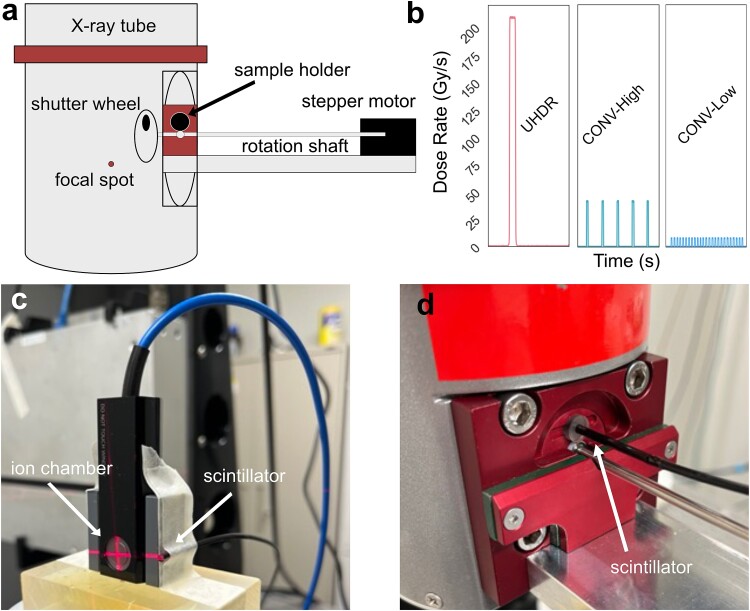
Diagram of the conventional X-ray tube fitted with shutter system (a). Scintillator measurements of each delivery time structure (b). Photographs of parallel plate ionization chamber and scintillator calibration procedure (c) and scintillator measurements within the shutter system (d).

The irradiations described in this work were conducted using a conventional X-ray tube modified with a shutter system to precisely control exposure times. This system has previously been characterized elsewhere [27, 32, 33]. For these experiments, the tungsten shutter wheel was updated with a 5.1-mm-diameter circular aperture as shown in Figs 1 and 2. This design functions by rotating the aperture into a fixed position for the duration of the exposure. The circular aperture allows for delivery of uniform dose distributions to a ∼5-mm-diameter area on the acrylic immobilization cylinder. For all irradiations, the tube voltage was set to 120 kVp. The instantaneous dose rate of the X-ray tube is directly proportional to the tube current. For UHDR irradiations, a single pulse at a tube current of 25 mA was used. For CONV treatments, tube current was reduced and additional pulses were delivered to reach the same total dose in a delivery time of ∼100 s, yielding average dose rates on the order of typical CONV treatments. The width of the pulse (exposure duration) was varied between 75 and 200 ms to deliver doses from 17 to 44 Gy. CONV delivery regimens consisted of either 5 pulses at 5 mA with 25-s inter-pulse spacing (referred to as CONV-High) or 25 pulses at 1 mA with 4-s inter-pulse spacing (CONV-Low) as shown in Fig. 1. The ability to linearly scale dose rate with tube current and dose with shutter exposure time has previously been demonstrated [33].

The dose to the surface of the acrylic immobilization cylinder was measured under the same operating conditions as the larvae irradiations with multiple dosimeters. Dosimetry was performed with ionization chambers, radiochromic film and plastic scintillator detectors (PSDs). Ionization chambers, which are the gold standard for measuring dose, cannot fit into the 6.5-mm-diameter sample holder where the larvae were irradiated. Instead, ionization chambers were placed outside of the shutter assembly and used for cross-calibration of smaller dosimeters (as shown in Fig. 2d). Calibrated radiochromic film was placed on the surface of the acrylic immobilization cylinder to measure the 2D dose distribution and the total dose delivered to larvae. PSDs, which emit optical light in response to ionizing radiation, were used to measure the response to X-rays at a sampling rate of 400 Hz. This high temporal resolution allowed for confirmation of the delivery time structure. Both film and PSDs were used to confirm the delivery of consistent doses at both UHDR and CONV dose rates. PSDs and films were calibrated with either a PTW 23342 parallel plate soft X-ray chamber (PTW, Freiburg, Germany) or PTW TN30013 Farmer chamber using the in-air method [34]. Laser cut EBT3 Gafchromic films (Ashland, Bridgewater, NJ) were placed on the surface of the acrylic immobilization cylinders providing a high-spatial-resolution 2D dose distribution measurement. Films were scanned 24 h post irradiation with an Epson flatbed scanner (Expression 10000 XL; Epson, Long Beach, CA). Pixel values were converted to dose using a red channel calibration curve generated with Farmer chamber measurements. PSDs connected to a hyperspectral dosimetry system, Hyperscint RP-100 (Medscint, Quebec City, QC), were used to measure light output from irradiations at a frame rate of 400 Hz.

### Statistical analysis

All statistical analyses were performed with Python 3.8. Mann–Whitney *U* tests and Student’s *t*-tests were performed to assess differences in eclosion between dose rate groups. Kaplan–Meier lifespan analysis was conducted using the lifelines package [35]. Results were regarded as significant for eclosion at *P* < 0.05. For lifespan analysis, log-rank tests were used and considered significant at *P* < 0.01.

## RESULTS

### Dosimetry

The X-ray shutter system produced uniform 2D dose distributions on the surface of the acrylic cylinders, allowing three larvae to be irradiated simultaneously. PSD and film dosimetry confirmed that doses delivered with UHDR and CONV agreed within 0.1%. High temporal resolution measurements with the PSD also confirmed the time structure of the delivered radiation, i.e. the pulse width and inter-pulse spacing. The average dose rates (over the duration of the treatment) were determined from film measurements: 210 Gy/s for UHDR and 0.2 to 0.4 Gy/s for CONV.

### Larva development

**Fig. 3:**
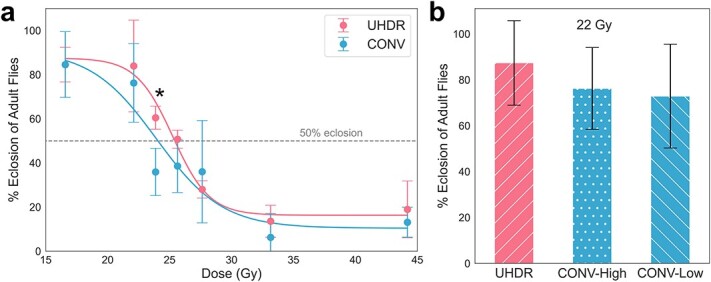
Eclosion (emergence as viable adult flies) irradiated with UHDR and CONV-High with doses from 17 to 44 Gy (a). Error bars show the standard deviation of three replicates (*n* = 60 for each dose). Comparison of eclosion rates from flies irradiated to 22 Gy with UHDR (1 × 25 mA), CONV-High (5 × 5 mA) and CONV-Low (25 × 1 mA) delivery regimens (b). ^*^Statistical significance between UHDR and CONV eclosion rates.

Post irradiation, larvae developed into pupae and then emerged as adult flies several days later. The rate of survival to adulthood decreased with dose as shown in Fig. 3 and described in Table 1. The average eclosion rate from all control groups was 86.1%. The overall dose-response curves presented in Fig. 3a do not differ significantly between UHDR and CONV (*P* = 0.841 from Mann–Whitney *U* test). However, at a dose of 24 Gy the mean emergence of adult flies was 60.5% for UHDR and 35.9% for CONV (*P* = 0.012 from Student’s *t*-test). From the sigmoidal fits of dose response, the *LD*_50_ was calculated to be 25.4 Gy for UHDR and 24.1 Gy for CONV. Five replicates were used for the 24-Gy dose groups (*n* = 100 for both dose rates).

**Table 1:** Fraction of irradiated larvae that eclosed as adult flies normalized to control

**Dose (Gy)**	**UHDR**	**CONV**	**UHDR/CONV**	***P*-value**
17	0.845	0.846	1.00	0.502
22	0.839	0.762	1.10	0.288
24	0.605	0.359	1.68	**0.012** ^*^
26	0.507	0.386	1.32	0.091
28	0.280	0.360	0.78	0.707
33	0.136	0.062	2.17	0.193
44	0.189	0.131	1.45	0.263

^*^Statistically significant differences between UHDR and CONV-High eclosion rates.

Additionally, eclosion rates for 22-Gy irradiations performed at UHDR and CONV-High and CONV-Low dose rates are presented in Fig. 3b. While the UHDR group showed higher eclosion, with 87.4% compared to 76.2% and 72.8% for CONV-High and CONV-Low, respectively, the differences were not statistically significant (*P* = 0.177 and 0.157). The mean eclosion rate for CONV-Low was 4% lower than CONV-High and was also not significant (*P* = 0.417).

**Fig. 4:**
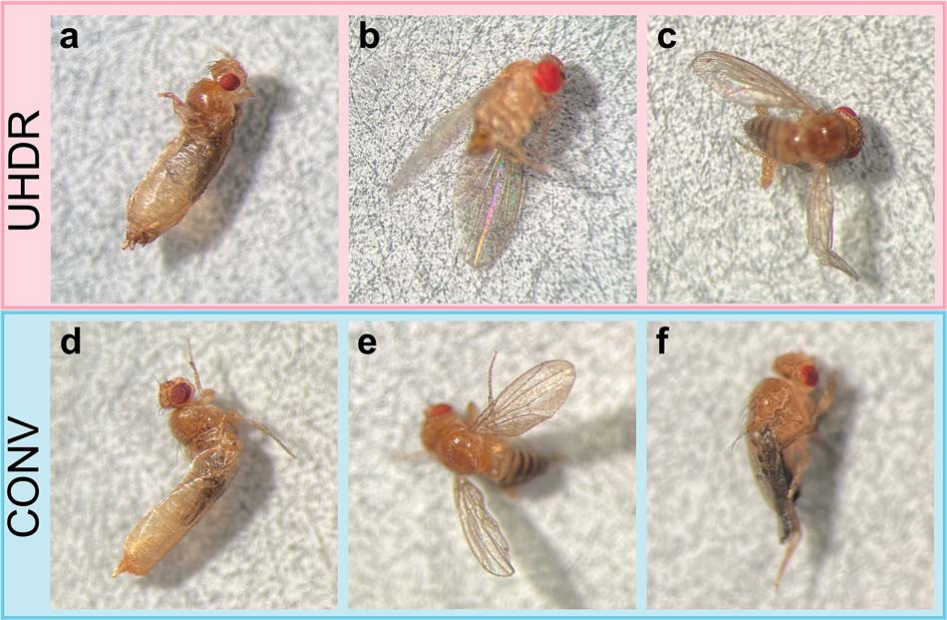
Emerged adult flies irradiated with UHDR and CONV X-rays at 24 Gy as larvae. Some flies from both dose-rate groups were unable to fully eclose from pupae (a, d). Radiation damage also leads to wing abnormalities, including failure to close normally (b, e) and deformity (c, f).

Irradiated *D. melanogaster* larvae that developed to adulthood showed several signs of physical radiation damage including severe deformity that prevented complete eclosion (which were not scored as emerged adults) and a range of wing malformations. These included wings that failed to completely open or close as well as partially developed wings. Examples of physical radiation damage to adult flies are shown in Fig. 4.

No offspring were found in vials holding adults irradiated with 26-Gy UHDR (and greater), and 24 Gy CONV (and greater) indicating sterility of adults, though conclusions about sex specific sterility cannot be reached. Larvae and pupae were found in vials containing adult flies irradiated with 24-Gy UHDR (and lower), 22-Gy CONV (and lower) and in all vials from control flies.

**Fig. 5:**
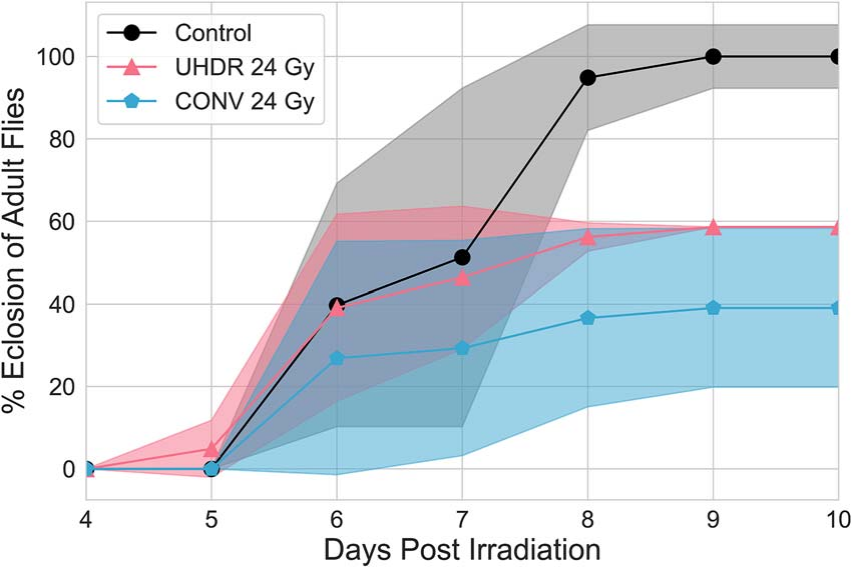
Time of emergence from pupal case of larvae irradiated with 24 Gy, normalized to the maximum eclosion of the control group. The shaded area indicates the standard deviation from three replicates.

Larvae irradiated with 24 Gy were scored daily for emergence from the pupal case. Figure 5 shows higher emergence of the UHDR irradiated flies compared to CONV at each day post irradiation. Emergence curves appear to be shifted vertically relative to the control group, indicating that irradiations are not inducing a delay in eclosion.

**Fig. 6:**
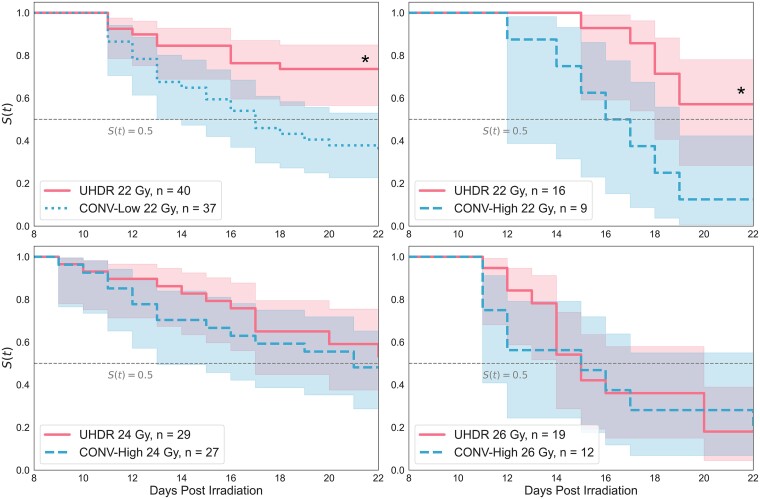
Kaplan–Meier survival estimator for emerged adults irradiated with 22, 24 and 26 Gy as larvae. The number of live adult flies included in the analysis is shown in the legend of each subplot. CONV-Low treatments are shown with a dotted line and CONV-High treatments are shown with a dashed line. ^*^Statistical significance between UHDR and CONV median lifespans.

Adult flies exposed to 22, 24 and 26 Gy as larvae were scored daily for survival up to 22 days post irradiation. The Kaplan–Meier estimator for probability of survival after irradiation is shown in Fig. 6 and the median survival times are summarized in Table 2. The median survival times were 17 days for flies treated with either CONV-High or CONV-Low at 22 Gy. The UHDR groups exhibited a median survival time >22 days at 22 and 24 Gy, and 15 days at 26 Gy. The probability of survival was higher for flies treated with UHDR at all time points post irradiation for both 22 and 24 Gy. The differences in log-rank survival was significant for 22 Gy for both CONV-High and CONV-Low treatments (*P* = 0.008 and 0.009).

**Table 2:** Median survival times post irradiation for adult flies treated with 22, 24 and 26 Gy

	**Median survival (days)**		
**Dose (Gy)**	**UHDR**	**CONV**	***P*-value**
22	*>*22	17	**0.009** ^*^
24	*>*22	21	0.286
26	15	15	0.735

^*^Statistical significance from log-rank test when *P* < 0.01.

## DISCUSSION

*Drosophila melanogaster* larvae irradiated with UHDR X-rays exhibited normal tissue sparing compared to CONV treatments in two scenarios. Statistically significant differences were observed for increased eclosion rates of larvae treated with 24-Gy UHDR and extended lifespan for flies irradiated with 22-Gy UHDR when they were compared to the respective CONV irradiations. These effects could be considered acute and long-term effects of radiation damage, which may have implications for determining dose limits in a radiotherapy context. Neither acute nor long-term sparing was observed for flies irradiated with doses *<*22 Gy or *>*26 Gy, indicating a possible threshold and limit for observing a FLASH effect given our study design. It is indeed expected that very low or very high doses would have similar responses regardless of dose rate. The magnitude of FLASH sparing has been shown to be dependent on dose, tissue type and the endpoint observed [36]. Previous studies have observed a threshold dose of ∼20 Gy for tissue sparing *ex vivo* and *in vitro* [12, 37]. Proton irradiations of zebrafish embryos have suggested a dose window for tissue-sparing effects ∼10–50 Gy [28]. The overall dose response and tissue-sparing effects described in Figs 3 and 6 should not be considered directly applicable to human tissues. Rather, this study identifies the dose and delivery time structure for two tissue-sparing effects in flies that could be exploited for information about the mechanism underlying the FLASH effect.

Interestingly, the two tissue-sparing effects did not overlap at the same doses. The differences in eclosion rate at 22 Gy were not statistically significant nor was the difference in lifespan for flies irradiated with UHDR and CONV at 24 Gy. The CONV treatments for 22 Gy were delivered with both CONV-Low and CONV-High regimens, both of which resulted in shorter median lifespans compared to the UHDR irradiated flies. For both treatments, the total irradiation time was kept the same, yielding a constant average dose rate of 0.2 Gy/s (delivered dose/total time). However, the number of pulses, inter-pulse spacing and instantaneous dose rate (governed by tube current) was changed to reach the same total dose. The similarity in lifespan between CONV-Low and CONV-High suggests that the difference in pulsed dose rates (8.8 Gy/s compared to 44.2 Gy/s) is less important for triggering the longer time scale normal tissue-sparing effects than the average dose rate over the duration of the treatment. CONV dose rates are typically defined as ∼0.1 Gy/s; however, dose rates within an order of magnitude have been shown to have similar radiobiological effects. For example, using a linac with and without a flattening filter, Nakano *et al.* showed that cell survival was unchanged when irradiated with dose rates spanning 0.05–0.33 Gy/s [38]. Other studies have shown UHDR normal tissue sparing in mice relative to CONV dose rates of 0.05 Gy/s [39], 0.079 Gy/s [6] and 0.9 Gy/s [40].

It should be noted that the median survival times calculated from the Kaplan–Meier estimator and reported in Table 2 suggest that flies irradiated with 24-Gy CONV survive longer than the 22-Gy CONV treatment. This is not indicative of a sparing effect from dose escalation and is in fact an artifact due to normalizing to the number of eclosed adult flies rather than the number of irradiated larvae. Note that fewer adults are included in the Kaplan–Meier analysis for 24 Gy than for 22 Gy shown in Fig. 6.

While this work represents the first UHDR irradiation study of *D. melanogaster*, there are a number of studies on the dose response of *D. melanogaster* at different life stages. Insects are significantly more radioresistant than mammals, and *D. melanogaster* are no exception. However, there are great inconsistencies in the literature. One study found that the minimum dose to cause lethal damage to third-instar larvae was 500 Gy, while the *LD*_50_ was 1429 Gy [41]. The *LD*_50_ reported varies widely depending on the definition of the endpoint used. For late-third-instar larvae, the *LD*_50_ is frequently cited as 4000 R [24]. Under the definition used in that work, this dose results in >95% emergence of adults from the pupal case followed by 50% mortality of adults at 48 h post eclosion. This description is inconsistent with the results presented here in terms of both emergence and survival of adult flies. It is also inconsistent with other recent works investigating the dose response to 0.662–6-MeV X-rays and 225-MeV protons, which found that the doses required to reduce eclosion to 50% was between 40 and 50 Gy [42, 43]. Furthermore, the use of low-energy X-rays in this work raises the issue of relative biological effect (RBE). Low-energy X-rays have been estimated to cause more biological damage per unit dose compared to standard megavoltage radiotherapy beams (RBE *>* 1). The dose-dependent RBE makes it difficult to directly compare the dose response of the flies in these experiments to other works. It is apparent that the focus on acute endpoints, including lack of mobility in larvae and 48-h mortality of adult flies, and inconsistency in endpoint definition has created confusion about the radiosensitivity of *D. melanogaster*.

The largest difference in emergence of adult flies was observed at 24 Gy, where UHDR had 68% greater emergence than CONV. This suggests that it may be possible to increase the therapeutic window by using UHDR radiotherapy for some intermediate doses. The reduction in normal tissue toxicities detailed here (eclosion rate and lifespan) is the first prerequisite in demonstrating a FLASH effect induced by low-energy X-rays. While the differential effect of UHDR and CONV dose rates on normal tissues is promising, iso-efficacy between UHDR and CONV on tumor tissues must be shown to establish a potential clinical benefit. *Drosophila melanogaster* is a promising model for understanding potential mechanisms of a FLASH effect, as the vast majority of human disease genes have homologues in *D. melanogaster* [44, 45]. In addition, the genetic and cellular bases of many processes connected to potential FLASH mechanisms, such as innate immune response [46], apoptosis and defense against reactive oxygen species, are well understood in fruit flies. Indeed, *D. melanogaster* is already well established as a model for studying cancer, with a vast array of methods for the study of fly tumors [47].

In addition to differences in survival and lifespan between UHDR and CONV, this work also revealed early signs that the effects of radiation on *D. melanogaster* fertility may be dose-rate dependent. While the surviving adults irradiated with UHDR yielded progeny at higher doses (up to 24 Gy) than the corresponding groups irradiated with CONV (up to 22 Gy), this experiment was not designed to provide quantitative fecundity results. Future experiments should isolate irradiated male and female flies and cross them with unirradiated virgin flies.

## CONCLUSIONS

*Drosophila melanogaster* larvae were irradiated with conventional and ultrahigh-dose-rate X-rays generated by a shutter-controlled 120-kVp X-ray tube. Larvae irradiated with 24 Gy at 210 Gy/s demonstrated 68% higher rates of eclosion compared to larvae irradiated at 0.24 Gy/s. Additionally, flies irradiated with 22 Gy had higher probabilities of survival post irradiation and longer median survival times, respectively, when irradiated with UHDR compared to CONV. Significant differences in eclosion or lifespan were only observed for flies irradiated with doses between 22 and 26 Gy, suggesting that there is a dose window to observe the FLASH effect in *D. melanogaster*. UHDR irradiation may decrease rates of radiation-induced sterility compared to CONV. The similarity of response between CONV-Low and CONV-High treatments suggests that average dose rate may be more important than instantaneous dose rate in triggering normal tissue-sparing effects.

## Supplementary Material

S1_rrae079

S2_rrae079
