# Supplementary figures and images for: High-throughput, low-cost FLASH: irradiation of Drosophila melanogaster with low-energy X-rays using time structures spanning conventional and ultrahigh dose rates
Source: J Radiat Res. 2024 Oct 18;65(6):836–44. doi: 10.1093/jrr/rrae079 (PMC11629999; doi:10.1093/jrr/rrae079)

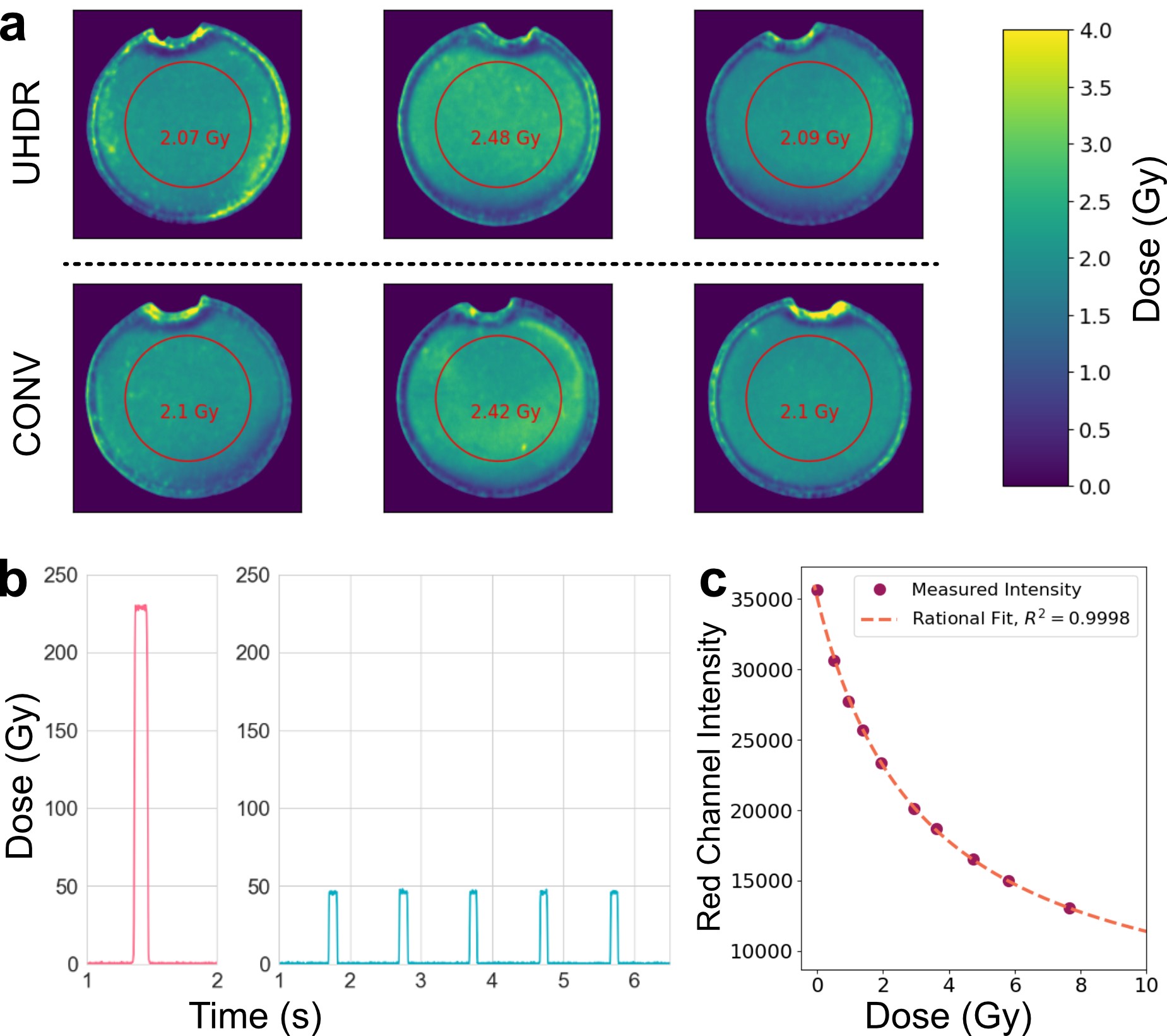

Supplement: S1_rrae079 [file s1_rrae079.jpeg]

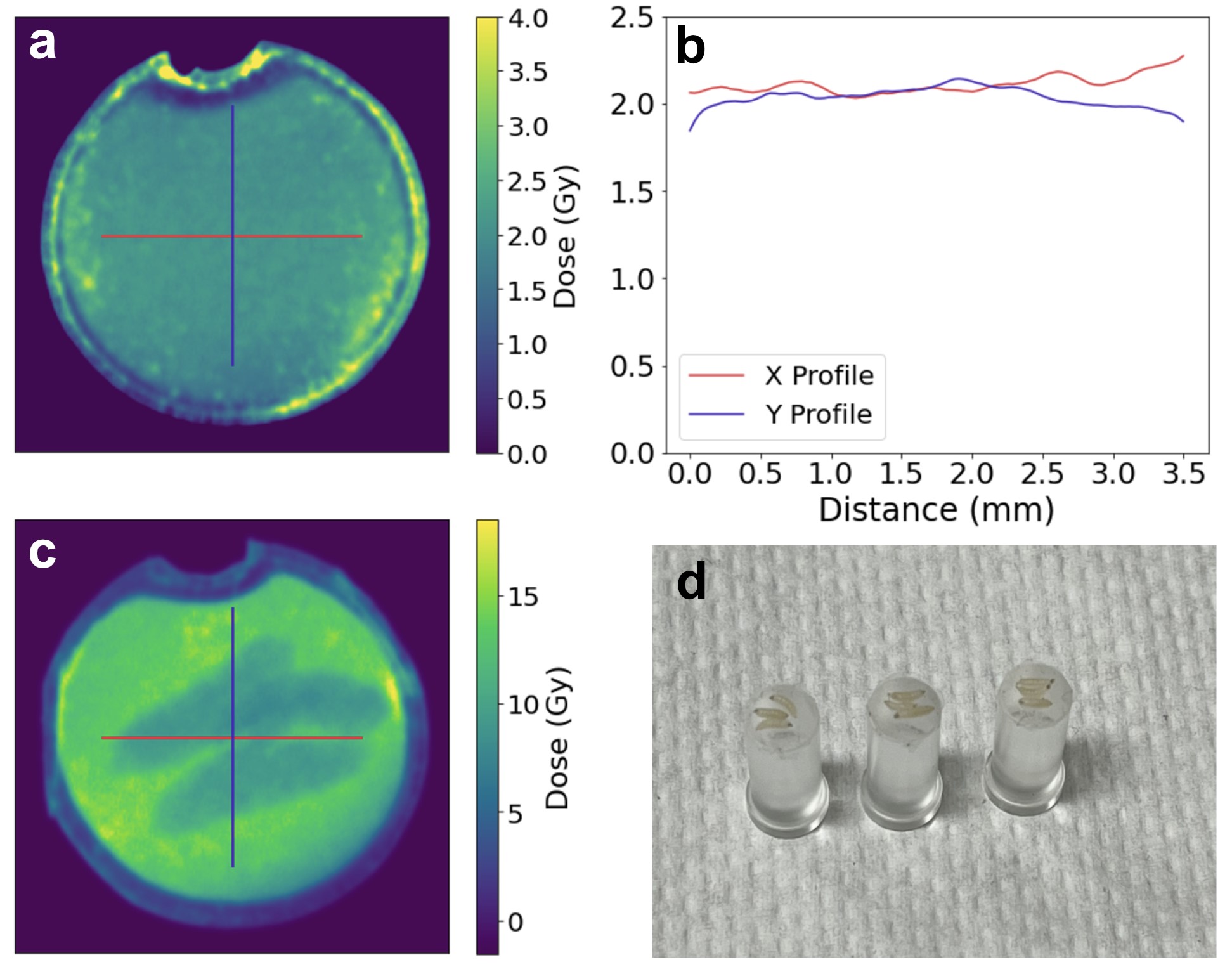

Supplement: S2_rrae079 [file s2_rrae079.jpeg]
